# Organizer activity in the mouse embryo^☆^

**DOI:** 10.1016/j.cdev.2025.204001

**Published:** 2025-02-05

**Authors:** Jenny Kretzschmar, Katharine Goodwin, Katie McDole

**Affiliations:** MRC Laboratory of Molecular Biology, Cambridge Biomedical Campus, Cambridge CB2 0QH, United Kingdom

**Keywords:** Organizer, Gastrula, Node, AVE, Mouse, Pre-chordal plate

## Abstract

The discovery of the embryonic organizer by Hilde Mangold and Hans Spemann in 1924 was one of the most ground-breaking achievements in the 1900 century for developmental biologists and beyond. Ever since the organizer was first described in newts, developmental biologists have been trying to uncover similar structures in other organisms. While the Spemann-Mangold organizer as an axis-inducing centre is evolutionary conserved in vertebrates, similar organizing centres have yet to be observed in mammals. In this review, we will provide a brief historical overview of the discovery of the mouse gastrula organizer and discuss its potential as an organizer throughout early post-implantation mouse development. We discuss cell migrations through the mouse organizer region and morphogenesis of organizer cells and tissues. Finally, we examine the evidence arguing for and against the existence of a head organizer in mice, and the role of the anterior visceral endoderm and the prechordal plate in organizing head structures.

## Abbreviations

AP: anterior-posterior
AVE: anterior visceral endoderm
EGO: early gastrula organizer
EMT: epithelial-mesenchymal transition
DE: definitive endoderm
DV: dorsoventral
GO: gastrula organizer
MET: mesenchymal-epithelial transition
MGO: mid-gastrula organizer
LGO: late gastrula organizer
PrCP: prechordal plate
VE: visceral endoderm

## 1 Introduction

The question of how the embryo develops from a single cell to a complex, 3-dimensional organism with diverse cell types has long fascinated biologists. In most animals, the transition from a group of undifferentiated cells to a layered embryo with distinct cell lineages and the beginnings of a body plan occurs via gastrulation. Gastrulation marks the emergence of the three germ layers (ectoderm, endoderm, mesoderm) and the establishment of the anterior-posterior (AP) and dorsal-ventral axes of the embryo. Decades of ongoing work have elucidated some of the mechanisms by which gastrulation is orchestrated in various model organisms. The earliest work, performed without the aid of genetic manipulations and the imaging capabilities of modern developmental biology, involved delicate transplantation experiments in which small, labelled pieces of embryos were surgically removed by hand and grafted onto different locations on host embryos (Beddington, 1981; Knoetgen et al., 2000; Lawson et al., 1991; Tam and Beddington, 1992; Selleck and Stern, 1991; Kintner and Dodd, 1991; Waddington, 1932; Beddington, 1994; Tam et al., 1997a; Hornbruch and Wolpert, 1986; Tam et al., 1997b; Kinder et al., 2001; Mangold, 1933; Spemann and Mangold, 1924). These experiments enabled researchers to determine how the labelled cells of the graft were incorporated into host tissues and how host tissues reacted to the presence of grafted cells.

The most famous of these (commemorated in this special issue) is the Spemann-Mangold experiment. Briefly, using newt embryos with different pigmentation, Hilde Mangold and Hans Spemann showed that grafting a small piece of the blastopore lip (the site where gastrulation begins) onto a host embryo induced an additional body axis that included both cells of the graft and cells of the host embryo (Spemann and Mangold, 1924). This experiment was preceded by that of Ethel Browne-Harvey, in which she demonstrated that grafting a small piece of Hydra onto a host induces a new body axis made of cells from the host (Browne, 1909). These seminal experiments, along with many others, helped to define three important biological concepts in the field: induction, competence, and organizers (Anderson and Stern, 2016; Arias and Steventon, 2018). **Induction** refers to the ability of a group of cells to direct the fate of the surrounding cells, while **competence** is the ability of cells to respond to inductive signals. The graft in the Spemann-Mangold experiment was capable of **induction** because it was transplanted onto **competent** tissues. This property, attributed to just a small population of cells, led to the concept of an **organizer**: a population of cells that can organize its environment to form a “secondary embryo” (Spemann and Mangold, 1924).

Organizers were subsequently found in several other model organisms and could induce secondary axes when grafted onto an embryo of an entirely different species. Further, subtleties in the nature of organizers became apparent, namely that an organizer could be made of multiple organizers - each responsible for its own piece of the body plan and therefore each acting in a different time and place (Anderson and Stern, 2016). Whereas the amphibian organizer is capable of inducing nearly complete secondary axes on its own, cell populations in other species that have been dubbed organizers appear to be less potent. In particular, it has been difficult to apply to concept of the organizer in its purest sense to the mammalian embryo (Arias and Steventon, 2018): the structure in mammals (and birds) that is most similar to the Spemann-Mangold organizer is the node, which in mice can only induce a partial duplication of the embryonic axis (Beddington, 1994).

Here, we first provide an overview of the foundational work examining organizer activity in the gastrulating mouse embryo (Section 2), which primarily occurred in the decades after the Spemann-Mangold experiment. We also examine cell migrations through the mammalian organizer and morphogenesis of organizer structures (Section 3). Moving beyond gastrulation, we discuss the role of organizers or organizing activity in specification of the murine head (Section 4).

## 2 Gastrula organizer

### 2.1 Gastrulation

Gastrulation is common to all vertebrates but begins with different initial conditions (e.g., embryo shape, number of cells, embryonic environment) and proceeds in different ways in each class of animal. Amphibian and bird embryos, for example, both initiate gastrulation with a very large number of cells (thousands in *Xenopus laevis* and chick) that undergo rearrangements to form the body plan (Arias and Steventon, 2018; Snow, 1981). Conversely, gastrulation of the mouse embryo starts with a much smaller number of cells (around 600 cells) and proceeds with massive amounts of cell proliferation (Arias and Steventon, 2018; Snow, 1981; Snow, 1977). These differences in size, as well as the inaccessibility of implanted mammalian embryos, meant that lineage tracing and the construction of fate maps occurred in amphibian and chick embryos long before it was feasible in mouse (Snow, 1981). Cell number and proliferation rates also affect the results of lineage tracing experiments, as progenitor zones in the mouse are much smaller compared to those in amphibian and bird embryos, and tend to be overlapping and more plastic (discussed in (Arias and Steventon, 2018)).

Advances in the ex vivo culture of post-implantation mouse embryos eventually enabled lineage tracing in intact embryos (Beddington, 1981; Beddington, 1982; Tam and Snow, 1980; Parameswaran and Tam, 1995). To explore the potency of embryonic ectoderm, radiolabelled cells were injected into embryos at the late primitive streak stage and examined after culturing to the beginning of somitogenesis (Beddington, 1981). The resultant chimerism revealed that the embryonic ectoderm is pluripotent at primitive streak stages. When both the origin and injection site (homotypic transplants) are located at the distal tip of the embryo, labelled cells incorporate into the trunk and posterior regions, including the midgut and notochord. Anterior injections of anterior endoderm cells show labelling in surface ectoderm and neurectoderm (Beddington, 1981), while posterior injections show labelling of the embryonic and extra-embryonic mesoderm (Beddington, 1982). Lineage tracing with LacZ-expressing cells showed that the lateral and posterior parts of the epiblast give rise primarily to mesodermal derivatives, with extra-embryonic mesoderm moving through the primitive streak first, followed by embryonic mesoderm (Parameswaran and Tam, 1995). These results all suggested that there were regional differences in embryonic ectoderm, but it was still unclear how or when these differences arise.

Later work using heterotypic transplants revealed that these regional differences in cell fate were not due to pre-existing differences in the epiblast. Epiblast (~20 cells) grafted onto different parts of the embryo took on the same cell fates as the native cells of the graft site (Beddington, 1982; Parameswaran and Tam, 1995). The only part of the epiblast with a preferred cell fate was the anterior embryonic ectoderm, which tended to form surface ectoderm and neural ectoderm (Beddington, 1982). This difference in behaviour of cells in the head region was observed in later experiments, and many aspects of anterior/head morphogenesis in the mouse remain a mystery (see Section 3). Overall, these experiments showed the importance of local control over cell fates and demonstrated plasticity in cell fates in the early embryo.

### 2.2 The node

**Fig. 1:**
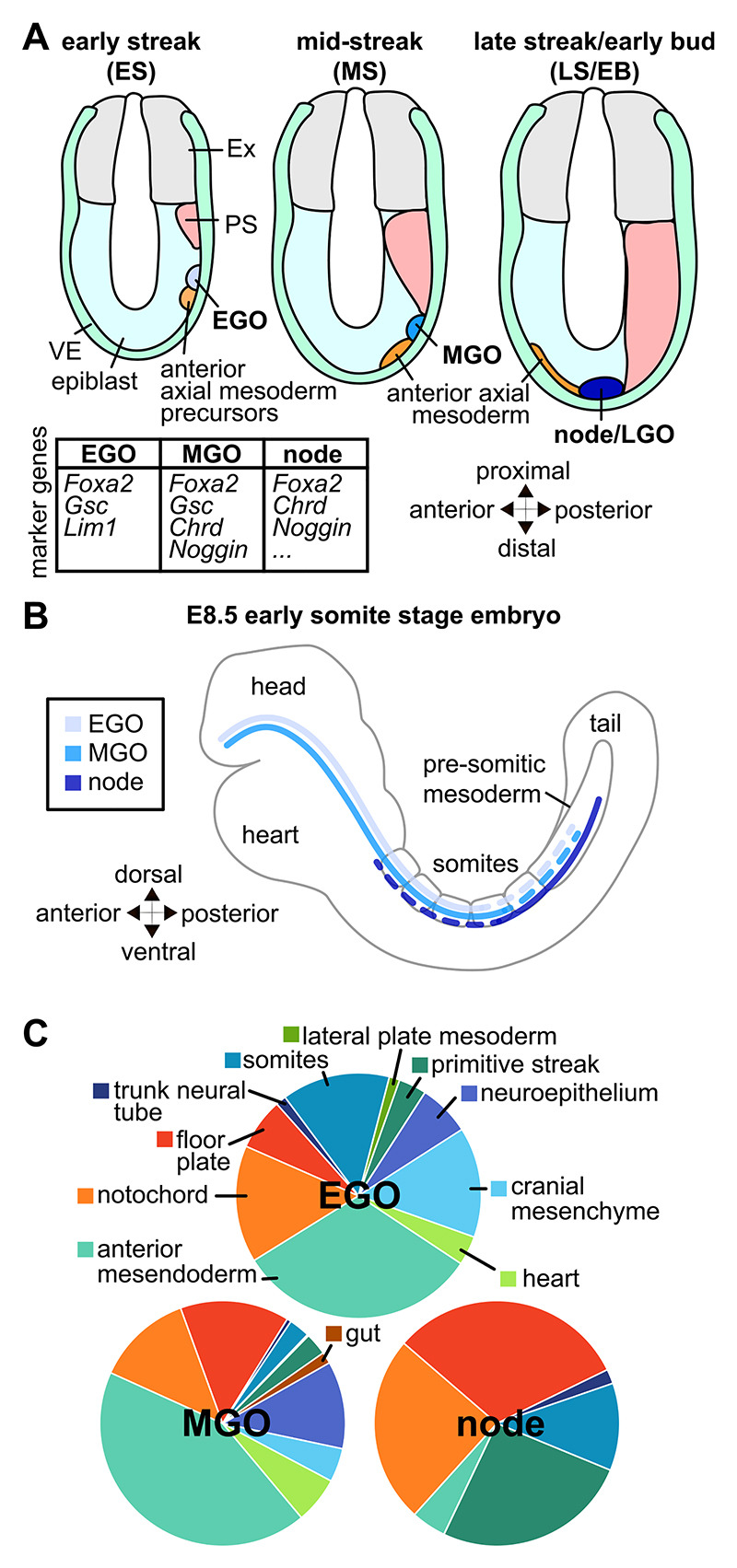
Gastrula organizer in the mouse embryo. (A) EGO, MGO, and node during gastrulation of the mouse embryo, including marker genes for each (for additional node markers, see (Davidson and Tam, 2000)). Early streak (ES), mid-streak (MS), and late streak/early bud (LS/EB) stages are shown, and relevant tissues are indicated: extraembryonic compartment (Ex), primitive streak (PS), visceral endoderm (VE), and anterior axial mesoderm. (B) Domains along the AP-axis containing cells of the EGO, MGO, and node in the early somite stage embryo (Kinder et al., 2001). Full line indicates greater contribution, dashed line indicates lesser contribution. (C) Fate of grafted cells of the EGO, MGO, and node at the early somite stage, from Table 1 of Kinder et al., 2001 (Kinder et al., 2001). Neuroepithelium refers to that of the forebrain and midbrain. Anterior mesendoderm includes the foregut endoderm and prechordal mesoderm. Gut refers to gut endoderm other than the foregut endoderm.

Given the similarities in body plans and gastrulation between the mouse and organisms with demonstrated organizers, it was generally agreed that there should be an organizer in the mouse (Lawson et al., 1991; Tam and Quinlan, 1996). Further, restriction of potency in the early mouse embryo seems to occur in a gradual, regional fashion throughout gastrulation (Lawson et al., 1991), in line with the idea of an organizer that moves along the axis of the embryo and changes its activity along the way. The likely candidate for the organizer in mouse was the node, a small structure at the distal tip of the late streak stage embryo (Fig. 1A). The murine node shares similarities with the dorsal blastopore lip of the amphibian embryo and Hensen’s node in the avian embryo both in terms of gene expression and developmental fates (Lawson et al., 1991; Selleck and Stern, 1991; Waddington, 1932; Blum et al., 1992; Hornbruch et al., 1979). While both amphibian and chick organizers had been shown to induce axis duplication upon transplant, similar experiments had not yet been carried out in the mouse embryo, likely due to the relatively small size of the mouse embryo and challenges in ex vivo culture. However, transplants onto embryos of other species supported the idea that the murine node acted as an organizer. Similar to Hensen’s node, the murine node induces digit duplication when grafted onto an avian limb bud (Hornbruch and Wolpert, 1986; Hogan et al., 1992). Transplanting the entire tip or just the node of a gastrulating mouse or rabbit embryo onto an early Xenopus gastrula induces the formation of anterior structures (Knoetgen et al., 2000; Kintner and Dodd, 1991; Blum et al., 1992).

Seventy years after the first publication of the Spemann-Mangold organizer, transplant experiments demonstrated organizing activity of the node in the mouse embryo (Beddington, 1994). Labelled nodes (~100 cells) transplanted onto posterior-lateral sites on host embryos could produce a secondary neural axis parallel to the host neural axis, including somites and neural folds (Beddington, 1994). In some cases, the transplanted cells would only incorporate into the host midline (Beddington, 1994). The donor cells contributed primarily to the notochord and the endoderm, and to a lesser extent, to lateral mesoderm and somites (Beddington, 1994). The duplicated notochords were made up exclusively of donor cells, without discernible recruitment of host cells, whereas the induced somites and neural folds were made mostly by host cells (Beddington, 1994). Histological analysis suggested that the induced neural folds resembled more caudal neural axis structures (i.e., spinal cord), rather than rostral (i.e., brain) (Beddington, 1994).

### 2.3 The early gastrula organizer

Given that node transplants failed to induce anterior-most tissues, it was possible that an earlier organizer, which eventually gave rise to the node, was responsible for patterning cranial structures. The earliest fate maps of the mouse embryo, based of single-cell horseradish peroxidase injections at pre-streak and early streak stages, showed overlapping domains of cells giving rise to the different germ layers (ectoderm, mesoderm, endoderm), extraembryonic tissue (extraembryonic mesoderm and amnion ectoderm), and of the head process and notochord (Lawson et al., 1991). In particular, at both the pre- and early streak stages, the small region at the anterior end of the primitive streak that gives rise to the notochord overlapped with those giving rise to ectoderm, endoderm, mesoderm and extraembryonic mesoderm (Lawson et al., 1991), suggesting a complex and multipotent population. This population therefore became a possible candidate for an earlier organizer.

Grafting experiments were then carried out to trace the lineage of these cells and test their organizing activity (Tam et al., 1992). Heterotypic transplants of labelled posterior epiblast cells onto the posterior of the embryo either incorporated into host tissues or induced axis duplication (Tam et al., 1992). In both cases, grafts produced tissues derived from the node (head process, notochord, notochordal plate, floor plate) (Tam et al., 1992). Some grafted cells found in the axial mesoderm were displaced along with midline extension, and migrated with the host mesoderm to end up in the cranial and axial mesenchyme (Tam et al., 1992). Grafts of early posterior epiblast thus recapitulated the axis duplication seen upon node transplantation, leading to this region being named the “early gastrula organizer” (EGO; Fig. 1A) (Tam et al., 1992). While the EGO could induce axis duplication, anterior structures were not properly duplicated (Tam et al., 1992; Tam and Steiner, 1999). Since the EGO was grafted onto the posterior of a gastrulating embryo, a possible conclusion was that posterior tissues are not competent to form anterior tissues. It was therefore possible that the EGO could instruct anterior patterning if grafted onto the correct location. However, transplants of the EGO onto the anterior of the embryo disrupted anterior development, leading to abnormal neural tube morphogenesis, the absence of neural folds and somites, and the formation of an anterior allantois (Tam et al., 1992). In this case, grafted cells remained in the anterior of the embryo, and axis duplication did not occur (Tam et al., 1992). Therefore, the EGO only shows organizing activity to a point, and is not equivalent to the Spemann-Mangold organizer.

### 2.4 The mid-gastrula organizer

While both EGO and node transplants could induce secondary axes, they expressed a slightly different set of organizer-related genes and contributed differently to derivatives of the axial mesoderm and other lineages (Fig. 1A-C) (Beddington, 1994; Tam et al., 1997a; Sulik et al., 1994; Camus and Tam, 1999; Davidson and Tam, 2000). These differences suggest that the EGO and node may correspond to different stages of the organizer, as had been observed in other species. Further transplant experiments examining the stages between the EGO and the formation of the node (i.e., mid-streak stage) identified an intermediate structure which was termed the mid gastrula organizer (MGO; Fig. 1A) (Kinder et al., 2001). The EGO and MGO contribute to roughly the same extent of the AP axis, but to a greater extent of the AP axis than the node, whose derivatives are found more caudally (Fig. 1B). The tissues populated by each stage of the gastrula organizer are also slightly different (Fig. 1C) (Kinder et al., 2001), consistent with a gradual change in organizer fates over developmental time and as the organizer moves along the AP axis.

## 3 Cell migration and morphogenesis in the organizer

The organizer is more than just a signalling centre – it is also a population of cells undergoing migration and morphogenesis. This includes the migration of the organizer itself and axial mesoderm cells through it, the migration of definitive endoderm (DE) cells through the organizer and their subsequent dispersal, and the morphogenesis of the node, prechordal plate, and notochord.

### 3.1 Migration of the organizer and axial mesoderm cells

**Fig. 2:**
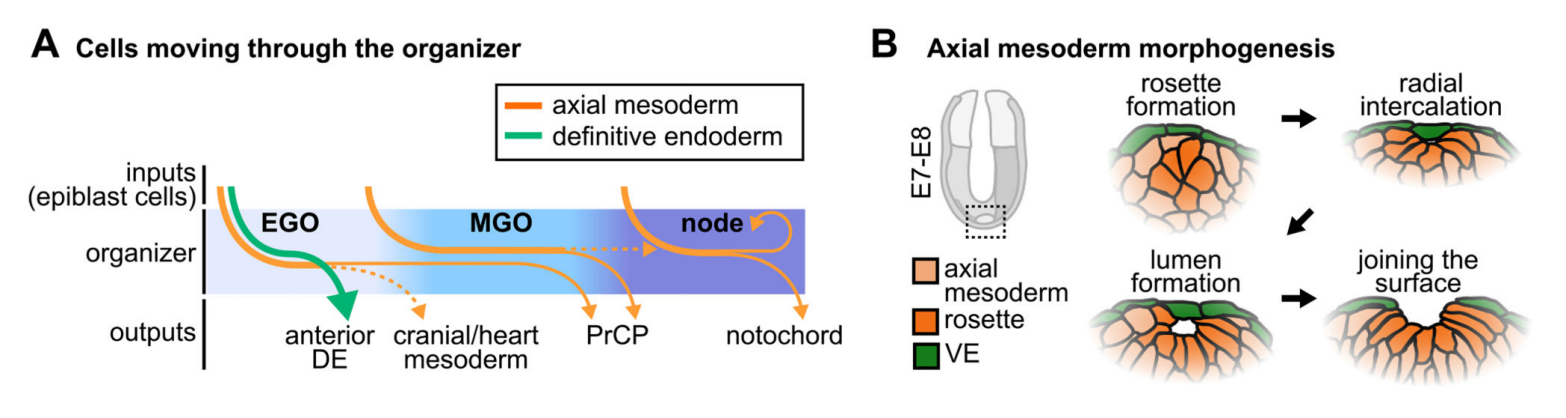
Cell migration and morphogenesis in the organizer. (A) Cells moving through the organizer at different stages. Inputs are cells from the epiblast near the organizer, including axial mesoderm and DE. Axial mesoderm outputs primarily contribute to the PrCP and notochord, with some cells of the EGO possibly contributing to cranial and heart mesoderm (derived primarily from the primitive streak, not the EGO). DE outputs give rise to the anterior endoderm. MGO cells contribute minimally to the node, suggesting that the cells of the node are instead recruited from the epiblast and then replenish the node or join the notochord. The exact timing and rates of inputs to and outputs from the organizer region are not known in detail, and, as such, this schematic simply illustrates the flow of cells through the organizer from early streak to late streak stages. (B) Axial mesoderm cells join the embryo surface via radial intercalation of multi-cellular rosettes from E7 to E8. Rosettes form beneath the surface, then radially intercalate outward and form lumens that eventually become continuous with the outside of the embryo.

The position of the organizer throughout gastrulation is at the anterior tip of the primitive streak. As the primitive streak expands, the organizer moves distally until the node is positioned at the tip of the embryo (Fig. 1A). During this distal movement, the EGO and MGO are not fixed structures of cells, but rather a region of the embryo with organizing activity that various progenitors occupy transiently. Similar cell behaviours have been observed in Hensen’s node in the chick embryo, where cells migrate through the node and only have organizing activity when they are within the confines of the node (Joubin and Stern, 1999). Tracking the movements of organizer cells was first accomplished using transplants of labelled cells onto unlabelled embryos from early to late streak stages (Kinder et al., 2001). The movements of EGO, MGO, and node cells could then be compared over time. EGO cells remain grouped together as they are moved distally/anteriorly by the elongation of the primitive streak, then eventually split into two groups: one remaining in the MGO, the other diverging into the surrounding mesoderm (Kinder et al., 2001). MGO cells migrate anteriorly along the midline until reaching the anterior margin of the neural plate, but were not found in the node (Kinder et al., 2001). Instead, cells proximal to the MGO were later found in the node and then eventually in the notochord (Kinder et al., 2001). This observation suggests that node precursors are absent from the MGO but incorporated into the gastrula organizer as it moves distally. Finally, cells of the node remain in the node as it retracts posteriorly, and eventually contribute to the posterior notochord (Kinder et al., 2001). Altogether, these experiments suggest that the mouse gastrula organizer is a zone that different progenitors pass through rather than a pool of pluripotent cells that produce each of the organizer-derived lineages (Fig. 2A). It is still unknown if and how the gastrula organizer is repopulated at different points in time, and improvements in spatial and temporal imaging resolution and reporter mice are needed to detail the exact times and locations of cell movement through the organizer.

### 3.2 DE cell migration through the gastrula organizer

In the early gastrula, DE cells migrate through the EGO region on their way to cover the anterior side of the embryo. Early labelling experiments showed the continuous emergence of DE cells from the embryonic ectoderm and through the anterior-most part of the primitive streak (Tam and Beddington, 1992). The first cells to emerge disperse widely towards the anterior and lateral surfaces of the embryo, eventually populating the endoderm around the future foregut. The axial mesoderm and this anterior DE lineage are specified early and separately in the epiblast (Probst et al., 2020). While both axial mesoderm and DE cells are specified from *Eomes*-expressing cells of the posterior epiblast, these two lineages come from spatially distinct *Eomes*+ populations: DE cells come from those at the anterior-most end of the primitive streak, whereas axial mesoderm cells come from those just proximal to this region (Probst et al., 2020). These two lineages are also specified at slightly different times, with mesoderm cells preceding DE cells (Probst et al., 2020). The finer movements of these first DE cells to emerge have not yet been described, so several questions remain: Do anterior DE cells pass through the organizer region, and/or do they contribute to the organizer population? Do anterior DE cells undergo epithelial-mesenchymal transition (EMT) as they move from the epiblast through the organizer region before migrating to the anterior side of the embryo? How does the emergence of anterior DE differ from that of later, posterior DE cells?

Mesodermal cells ingressing from the epiblast through the primitive streak undergo an EMT, and it was thought that this was also true for DE cells, requiring a subsequent mesenchymal-epithelial transition (MET) (Viotti et al., 2014). However, recent work showed that DE cells do not undergo an EMT-MET cycle, and that they are formed independently of the key EMT transcription factor Snail (*Snai1*) (Scheibner et al., 2021). Instead, they undergo an incomplete EMT, characterized by simultaneous expression of the mesenchymal and epithelial cadherins, N-cad-herin (*Cdh2*) and E-cadherin (*Cdh1*), and a transient downregulation of polarity, tight junction, and adhesion proteins (Scheibner et al., 2021). *Foxa2* expression in DE cells suppresses EMT molecular programs and *Snai1* expression and induces the expression of Wnt inhibitors to ensure DE cells retain an epithelial identity (Scheibner et al., 2021). These findings provide us with more detailed information about the establishment of this lineage and clues about the physical mechanisms by which DE cells leave the epiblast to populate the surface of the embryo.

### 3.3 Node and notochordal plate morphogenesis

Contrary to the EGO and MGO, the node is a morphologically distinct structure that can be identified visually when it protrudes from the distal tip of the embryo at the late bud/early headfold stages of mouse embryo development around E7.5 (Sulik et al., 1994; Downs and Davies, 1993). Cells expressing *Noto* (a marker for node and notochord) that eventually form the node first assemble beneath the surface of the embryo at the early bud stage (Yamanaka et al., 2007). As they join the surface layer, an indentation forms at the centre of the node, producing a horseshoe-like shape by the late bud-early headfold stage (Yamanaka et al., 2007). The cells around the periphery are called “crown cells”, and together with the indentation give the node its “crown and pit” morphology (Sulik et al., 1994; Yamanaka et al., 2007). This entire structure disappears by the 7–8 somite stage (Yamanaka et al., 2007). Organizing activity may require the entire, intact node, as smaller grafts (~20 cells) that have been enzymatically digested do not induce secondary neural axis formation, whereas intact node grafts (~100 cells) do (Beddington, 1981; Beddington, 1994; Beddington, 1982). Further, the structure of the node is important for its function in left-right patterning (Grimes and Burdine, 2017). Cells within the node are decorated with motile cilia that generate unidirectional fluid flow across the node, and impairment of these cilia randomizes left-right patterning (Nonaka et al., 1998). The “crown and pit” morphology of the node is thought to trap fluid, allowing pit cells to generate flow that crown cells respond to (Nonaka et al., 1998; Tabin and Vogan, 2003; Okada et al., 2005; Hirokawa et al., 2006; Tanaka et al., 2023).

The emergence of node and notochordal plate cells occurs gradually, with the small apical surfaces of these cells appearing between the more squamous visceral endoderm (VE) cells covering the embryo (Lee and Anderson, 2008). Over time, these cells form coherent layers on the surface of the embryo, perhaps in part due to coordinated removal of endodermal cells by migration or cell death (Lee and Anderson, 2008). In epiblast-specific knockouts of Lhx1, fewer axial mesoderm and DE cells reach the surface of the embryo, and displacement of overlying VE cells does not occur (Costello et al., 2015). It is unclear whether the displacement of VE cells is an active process, in which VE cells respond to an unknown signal and migrate out of the area, or a passive one, in which VE cells are displaced by the arriving axial mesoderm and DE cells. If it is an active process, then these epiblast-specific Lhx1 mutants, where VE cells are genetically wildtype, may lack the signal that causes VE cells to move out of the way. Instead, if it is a passive process, then failure of VE cell displacement may simply be due to a defect in axial mesoderm and DE migration to the surface.

Recent work has elucidated some of the cellular mechanisms driving the emergence of node and notochord cells. Prior to joining the endoderm on the surface of the embryo, notochord cells undergoing MET form rosette structures and then Par3-dependent apical domains (Gredler and Zallen, 2023). These apical domains form lumens, and rosettes coalesce to produce connected lumens as these collective structures radially intercalate and eventually incorporate into the surface endoderm layer (Gredler and Zallen, 2023). In Par3 mutants, notochord cells still perform MET and incorporate into the surface endoderm, albeit less efficiently and without forming a continuous node and notochordal plate on the embryo surface (Gredler and Zallen, 2023). Par3 mutants exhibit growth defects, a shorter embryonic axis, and defective heart development (Gredler and Zallen, 2023; Hirose et al., 2006), but it is not known whether these phenotypes are a result of altered morphogenesis of the node and notochordal plate, as opposed to other, local functions of Par3. Cranial-caudal axis organisation resulting from organizer activity prior to node emergence appear to be intact in Par3 mutants, whereas the caudal end of the axis is less developed (Hirose et al., 2006). Proper emergence of the node and notochordal plate may therefore be important for later events in patterning the caudal end of the axis.

## 4 Is there a head-organizer in mice?

Spemann observed that the axis-inducing properties of the dorsal lip in *Xenopus* change throughout gastrulation. While the transplantation of the dorsal lip region at the early gastrula stage resulted in the induction of a complete secondary axis including head structures, transplantation of these structures at a later stage resulted in the formation of a secondary axis lacking head structures (Spemann, 1931). Slightly later, Otto Mangold observed that grafts of the archenteron roof and medulla induce different tissues depending on the positional origin of the graft. While rostral-most grafts induce head structures, caudal-most ones induce tail structures (Mangold, 1933). These observations led to the hypothesis that either the morphogenetic properties of organizer structures change over time or that several distinct organizers induce the different regions of the body separately. In mice, the competence of organizer structures to induce head structures varies greatly throughout development. While transplantation of the node and EGO results in axis duplication, neither can produce head structures (Beddington, 1994; Tam et al., 1997a). Interestingly, transplanting the entire tip or just the node of a gastrulating mouse embryo onto an early Xenopus or chick gastrula induces the formation of anterior structures, including neural tissues (Knoetgen et al., 2000; Blum et al., 1992). This may be due to differences in the competence of the host tissue, leading to apparent organizer activity that does not represent how patterning actually occurs in the mouse embryo (Arias and Steventon, 2018). Therefore, in mice, the node might only function as a trunk organizer, like the late gastrula organizer in amphibians, suggesting that there may be a separate head organizer in the mouse embryo. This idea is also supported by the fact that embryos exhibit remarkably normal AP patterning even in null mutants of Hnf-3b (*Foxa2*) which lack a distinctive node (Ang and Rossant, 1994).

### 4.1 Anterior visceral endoderm

**Fig. 3:**
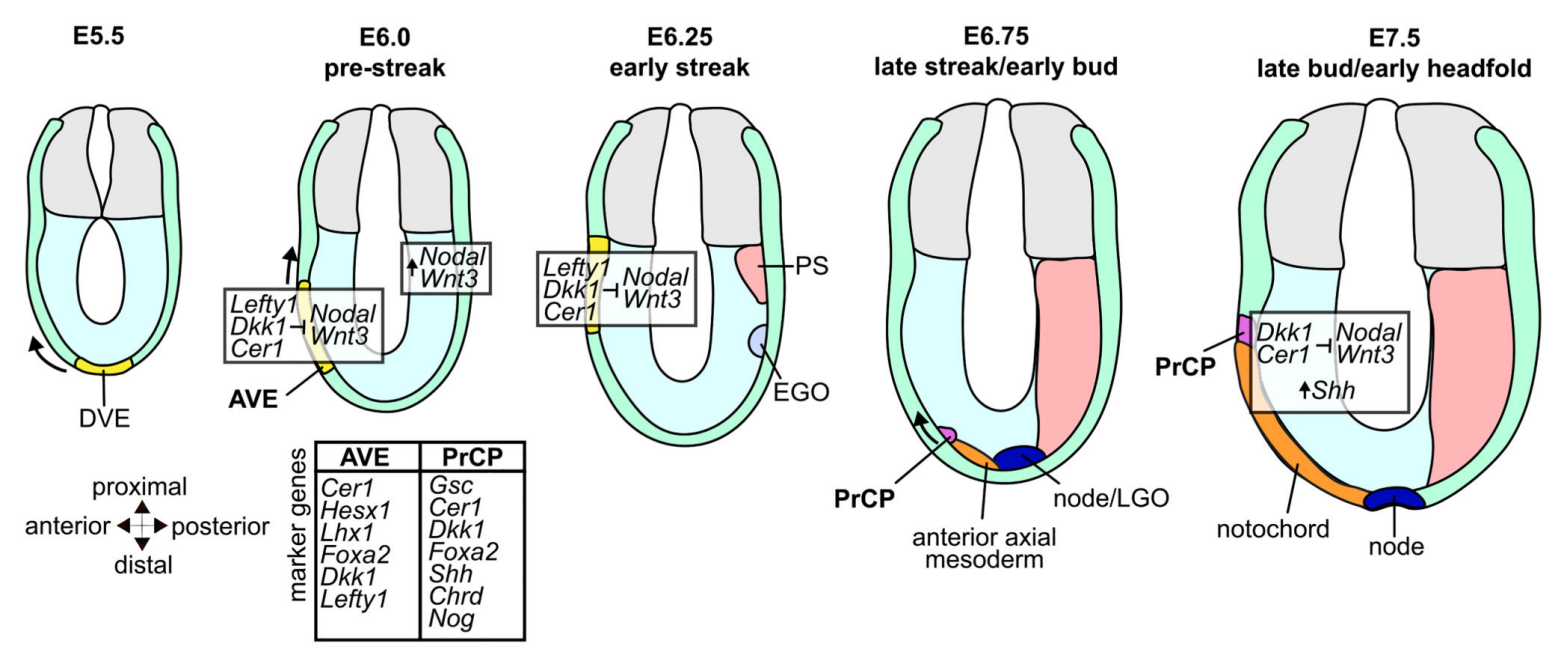
The AVE and PrCP in the formation of the head in mouse. At E5.5 distal visceral endoderm starts to migrate towards the anterior side, and is then referred to as the anterior visceral endoderm (AVE). The AVE expresses Nodal and Wnt antagonists, leading to the restriction of the primitive streak (PS) formation to the posterior at E6.25. After the complete formation of the PS, the anterior tip of the notochord gives rise to the prechordal plate (PrCP) at E6.75. The PrCP migrates towards the anterior side, expressing Wnt and Nodal antagonists as well as *Shh*. This leads to further stabilization of the anterior fate, controls the formation of head structures and the regionalization of the developing brain. The direction of migration is indicated by arrows.

One frequently discussed candidate for the anterior organizer role in the mouse embryo is the anterior visceral endoderm (AVE), which is formed at E5.5 at the most distal tip of the embryo. This patch then migrates anteriorly and proximally before finally residing just under the anterior extra-embryonic boundary (Fig. 3). The AVE expresses several genes associated with anterior patterning activity before primitive streak formation, including *Otx2, Lhx1, Gsc, Cer1*, and *Hesx1* (Shawlot et al., 1998; Thomas et al., 1998; Acampora et al., 1995; Belo et al., 1997; Thomas and Beddington, 1996). Smad2 mutants do not express *Cerl, Hesx1*, and *Lhx1* in the AVE, and do not express *Otx2* at early gastrulation stages (Waldrip et al., 1998), indicating a failure in the establishment of an AP axis. Removal of anterior endoderm cells expressing *Hesx1* during the early stages of gastrulation decreased *Hesx1* expression in the neuroectoderm and ultimately results in failed fore-brain (Thomas and Beddington, 1996). Taken together this indicates that the anterior identity of the mouse embryo might be established even before the onset of gastrulation. (Beddington and Robertson, 1998) and that signalling required for anterior patterning is specifically present in the anterior endoderm prior to the formation of the gastrula organizer.

There is also clear evidence against the role of the AVE as head organizer. *Dkk1* mutants fail to express *Hesx1*, resulting in malformation of the head. However, embryos with AVE-specific knockout of *Dkk1* show no abnormalities in their anterior organisation, indicating that *Dkk1* expression specifically in the AVE is not essential for the specification of head structures (Mukhopadhyay et al., 2001). Subsequently, it was shown that *Hesx1*, acting downstream of *Dkk1* and essential for forebrain morphogenesis, is autonomously required in the anterior neuroectoderm for normal forebrain formation and restricted knock-down of *Hesx1* expression in the AVE does not affect forebrain development (Martinez-Barbera et al., 2000). Furthermore, while the expression of *Cer1* is sufficient to induce ectopic head formation in Xenopus (Bouwmeester et al., 1996), *Cer1*^−/−^ mouse embryos have normal expression of *Otx2* and display no abnormalities in head morphogenesis (Shawlot et al., 2000; Simpson et al., 1999). These data suggest that different or multiple functionally redundant factors might control head formation in mouse.

Joint transplantation experiments of the AVE and the EGO suggest that it is the synergistic interaction of these two populations which controls successful anterior patterning. While transplantation of the EGO or AVE alone is not sufficient to induce the formation of ectopic neuronal tissue, joint transplantation is (Tam and Steiner, 1999) There is no question that the AVE has an important signalling function ensuring the correct pattering of head structures and overall anterior organisation, but its categorisation as an organizer by the generally accepted definition remains debatable. Furthermore, multiple genes expressed in the AVE that characterise and refine anterior identity are also expressed in the axial mesoderm and definitive endoderm after they emerge from the primitive streak. This brings up the possibility that the anterior patterning activity of the AVE is redundant in the presence of a normal node and axial mesoderm.

The role of the AVE lies therefore in restricting posteriorisation rather than actively inducing anteriorisation/head structures. The AVE actively antagonizes primitive streak formation by expressing nodal and Wnt antagonists (*Lefty1, Dkk1, Cer1* (Belo et al., 1997; Meno et al., 1998; Zakin et al., 2000; Perea-Gomez et al., 2001)), thus restricting the expression of nodal and Brachyury to the posterior side. Simultaneous mutation of *Cer1* and *Lefty1* results in the formation of multiple and incorrectly patterned primitive streaks, highlighting the importance of the expression of Nodal antagonists in the AVE to the establishment of the AP axis. *Cer1* and *Lefty1* are specifically needed in the AVE and act redundantly as only mutation of both genes simultaneously is sufficient to induce this phenotype. (Perea-Gomez et al., 2002) Additionally, the maintenance of extracellular matrix components like laminin and collagen IV by the AVE might play a crucial role in restricting EMT and mesoderm induction to the posterior epiblast (Egea et al., 2008).

### 4.2 The prechordal plate

Another structure debated as having potential head organizer activity in mice is the prechordal plate (PrCP). The prechordal plate originates from the most anterior part of the axial mesoderm and expresses organizer-marker genes, like *Gsc* (Blum et al., 1992) and *Dkk1* (Mukhopadhyay et al., 2001) at the early headfold stage.

During early development, the axial mesoderm and its derivatives are the main source of sonic hedgehog (*Shh*). (Fig. 3) *Shh* is involved in the establishment of the AP axis as well as the dorsoventral axis of the spinal cord and brain (Lumsden and Graham, 1995). Loss of *Shh* expression results in disrupted midline formation and neural tube closure defects. While *Shh* mutant embryos form a notochord, they fail to maintain it, and the notochord progressively loses Brachyury expression. Furthermore, *Foxa2* expression in the axial mesoderm is absent, suggesting that *Shh* expression from the notochord is required for HNF-3beta induction and normal neural tube development. (Chiang et al., 1996) Surgical removal of the PrCP recapitulates the phenotype of *Shh*^−/−^ embryos, showing that it is specifically the presence of *Shh* expression in PrCP cells that is required for midline development and organisation of the forebrain (Aoto et al., 2009). The same is true for *Dkk1. Dkk1* is expressed in the PrCP as well as in the anterior endoderm, but analysis of chimeric embryos with perturbed expression of *Dkk1* in only the AVE revealed that head induction relies on the expression of *Dkk1* exclusively in the PrCP (Mukhopadhyay et al., 2001).

The ENU-induced mouse mutation *nearly headless* (*nehe*) disrupts the formation of the PrCP, while the formation of other structures controlling forebrain formation and anterior organisation, like the AVE, anterior DE, and posterior axial mesoderm, display no abnormalities (Zhou and Anderson, 2010). This defect in the formation of the PrCP, characterized by the absence/reduction of the expression of *Foxa2, Gsc*, and *Dkk1* in the anterior midline, results in loss of anterior neural progenitors and forebrain structures (Zhou and Anderson, 2010). Further investigations demonstrated that this phenotype is the result of disrupted mitochondrial metabolism, caused by a partial loss of the lipoic acid synthetase (*Lias*), indicating that the formation and organisation of the PrCP is dependent on high levels of ATP (Zhou and Anderson, 2010).

While the genes expressed in the AVE and the PrCP point to a possible role for these tissues as head organizers, the precise pathways and mechanisms that specify the anterior in the mouse embryo are still not completely understood. Anterior specification in mouse is most likely an interplay of signalling activity from various sources, namely the AVE and the PrCP but also the anterior DE (ADE). After induction of the anterior neural fate by the AVE, this state is labile until signalling from the ADE and PrCP reinforce its specification (Barbera et al., 2000; Robb and Tam, 2004), and removal of the ADE and PrCP causes defects in forebrain development (Camus et al., 2000). Like in the AVE, the expression of BMP and Wnt signalling inhibitors in the AVE and PrCP most likely allow anterior specification and restrict posteriorsation.

## 5 Conclusions and perspectives

While it does not appear that the mouse has an equivalently potent or distinct organizer like the Spemann organizer in amphibians, mice have multiple sites exhibiting organizer activity, each with distinct abilities to influence neighbouring tissues and direct patterning (Fig. 1A, Fig. 3). As previously discussed in the context of the gastrula organizer, the capacity to induce neighbouring tissues varies throughout development as does the expression of genes associated with patterning activity. The patterning ability of all organizers across species is driven by homeo-domain proteins and transcription factors, which regulate key downstream pathways. The majority of these transcription factors target the canonical Wnt, Nodal, and FGF pathways, which are critical for coordinating embryonic patterning (reviewed in (Kumar et al., 2021)). Similar molecular profiles are seen in regions with patterning activity in mice, such as the gastrula organizer and the AVE. These organizer-like cell populations are characterized by the expression of homeodomain proteins like *Gsc* and *Foxa2*, and AP patterning by the AVE and PrCP relies on the expression of BMP, Nodal, and Wnt signalling inhibitors. However, the coordination of patterning is not solely based on the presence of individual pathway components but rather based on the tight regulation and interplay of these signalling pathways. Furthermore, it has been shown that the induction of organizer structures in *Xenopus laevis* explants can be controlled by mechanical cues (Bubna-Litic et al., 2024), highlighting another potential player that might influence the formation of organizer structures in mice.

The knowledge acquired about the molecular players and pathways involved in the establishment of the body axes of the mouse embryo has provided a useful toolbox for further exploration of how patterning is controlled in mammals and how it can be induced ex vivo, for example, in the context of 3D tissue culture. A remarkable example of this is the recent development of gastruloids (van den Brink and van Oudenaarden, 2021). Targeted manipulation of the above-mentioned signalling path-ways alongside specialized culture conditions allows the formation of 3D structures from stem cells, recapitulating gastrulation, including the formation of multiple derivatives of the three germ layers as well as axis formation. Experimental engineering of the above-mentioned morphogen signalling centres allows complex embryoids resembling many features of the neurula-stage mouse embryo, including the formation of all three germ layers, and the formation of an anterior-posterior patterned neural tube and gut tube (Xu et al., 2021). More recently, the use of self-assembling, synthetic organizer cells allowed a more targeted manipulation of morphogen gradients in embryonic stem cell cultures (Yamada et al., 2024). The generation of spatially and temporally defined morphogen gradients enabled the formation of more precisely patterned embryoids, resembling the patterning observed in mouse embryos in greater detail. As of yet, however, gastruloids still are not able to fully reproduce embryonic structures or both axes simultaneously, and form either posterior or anterior identities.

Our understanding of the organizing properties of the mouse embryo also provides a starting point for understanding how patterning might be controlled in experimentally less accessible organisms like primates or humans. Experiments with hESCs have shown that activating the TGFβ and WNT pathways is sufficient to induce the expression of organizer genes like *Gsc, Cer1*, and *Chrd*. Subsequent transplantation of these cells into a frog gastrula (Sharon et al., 2011) or chicken embryo (Martyn et al., 2018) results in the formation of a secondary axis, similar to the grafting experiments of Spemann and Mangold (Spemann and Mangold, 1924). These experiments highlight potential similarities between the induction of organizing structures in human and other vertebrates like the mouse.

Why then, is there no definitive mammalian organizer as there are in other species? This could potentially be due to differences in the rate of development between organisms, with mammals taking substantially longer to reach and complete gastrulation than amphibians. The number of cells in the mouse embryo at the start of gastrulation is also much lower than other species with well-defined organizers, and the mouse furthermore resides inside of a maternal environment that provides signalling factors of its own (Arias and Steventon, 2018; Snow, 1981). The gradual adoption of cell fates in the mouse aligns with the idea that the mouse organizer also changes over the course of development, regionalizing and specifying along with the tissues and cells it influences. Experimental induction of a complete embryonic axis in mammals, including anterior and posterior tissues, may then require coaxing an organizer population by dynamically supplying signalling factors and allowing it to differentiate in situ with the host tissues. This specific spatiotemporal tuning of organizer activity will require a deeper investigation in the changing molecular network of the mouse organizer (s), and precise genetic control over the many signalling pathways involved.

## Data Availability

N/A.
